# Memristive Characteristic of an Amorphous Ga-Sn-O Thin-Film Device with Double Layers of Different Oxygen Density

**DOI:** 10.3390/ma12193236

**Published:** 2019-10-02

**Authors:** Ayata Kurasaki, Ryo Tanaka, Sumio Sugisaki, Tokiyoshi Matsuda, Daichi Koretomo, Yusaku Magari, Mamoru Furuta, Mutsumi Kimura

**Affiliations:** 1 Department of Electronics and Informatics, Faculty of Science and Technology, Ryukoku University, Seta, Otsu 520-2194, Japan; 5krskayt-ryu.fc@ezweb.ne.jp (A.K.); wesker16.barus@icloud.com (R.T.); sumio.suzi@gmail.com (S.S.); 2 Innovative Materials and Processing Research Center, High-Tech Research Center, Ryukoku University, Seta, Otsu 520-2194, Japan; mazda.toki@gmail.com; 3 School of Environmental Science and Engineering, Graduate School of Engineering, Kochi University of Technology, Kami, Kochi 782-8502, Japan; 216003c@gs.kochi-tech.ac.jp (D.K.); 216007n@gs.kochi-tech.ac.jp (Y.M.);; 4 Division of Information Science, Graduate School of Science and Technology, Nara Institute of Science and Technology (NAIST), Takayama, Ikoma 630-0192, Japan

**Keywords:** memristive characteristic, amorphous Ga-Sn-O (α-GTO), thin-film device, oxygen density

## Abstract

We have found a memristive characteristic of an amorphous Ga-Sn-O (α-GTO) thin-film device with double layers of different oxygen density. The double layers are deposited using radio frequency (RF) magnetron sputtering, whose gas for the lower layer contains less oxygen, whereas that for the upper layer contains more oxygen, and it is assumed that the former contains more oxygen vacancies, whereas the latter contains fewer vacancies. The characteristic is explained by drift of oxygen and is stable without forming operation because additional structures such as filament are unnecessary. The fabrication is easy because the double layers are successively deposited simply by changing the oxygen ratio in the chamber.

## 1. Introduction

Amorphous metal-oxide semiconductor (AOS) thin-film devices are broadly employed as thin-film transistors (TFTs) [1,2,3,4,5,6,7,8,9,10,11] in flat-panel displays (FPDs) [12], such as light-emitting diode displays (OLEDs) [13] and liquid-crystal displays (LCDs) [14], because they have high performance, excellent stability [15,16], and easy manufacturability [17,18]. AOS thin-film devices are also promising to various applications, such as computing units [19,20], power devices [21,22], and thermoelectric devices [23,24], because specific characteristics can be obtained for individual requirements by customizing materials, structures, fabrications, etc. They can be fabricated at a low temperature on a large area for a low cost. Particularly, we are focusing on amorphous Ga-Sn-O (α-GTO) thin-film devices not only for TFTs [25,26] and thermoelectric devices [27], but also for neuromorphic systems [28,29]. The α-GTO thin-film devices do not include rare metals, such as In, and industrial issues on supply anxiety and resource depletion can be solved. Ga-based technology is currently expensive because the extraction technique is expensive. Since the abundance in Earth’s upper continental crust of Ga is eight times more than that of In [30], Ga-based technology may be inexpensive in the future.

Memristors are passive devices with electrical conductance depending on the past history of the electrical current [31], and they have been recently employed for resistive random access memory (ReRAM) [32], neural networks [33], etc. However, the conventional memristors require expensive elemental materials, device structures, manufacture processes, etc. For example, Hf and Pt [34], multiple layers of different materials [35], and precisely controlled manufacturing processes to get stoichiometric and nonstoichiometric crystals [36] are used. Recently, we discovered a memristive characteristic of an α-GTO thin-film device, by which the abovementioned problems were solved [37]. However, the initial several ten cycles were necessary until the memristive characteristic became stable.

In this study, we found a memristive characteristic of an α-GTO thin-film device with double layers of different oxygen density. In comparison with the previous results, the memristive characteristic is stable even from the initial hysteresis. In this paper, the device structure, memristive characteristic, and repetition characteristic of the α-GTO thin-film device will be shown, and the operating mechanism of the memristive characteristic will be discussed.

## 2. Materials and Methods

**Figure 1:**
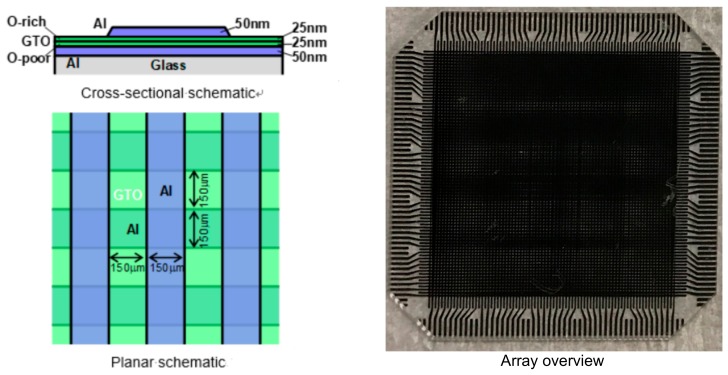
Device structure of the amorphous Ga-Sn-O (α-GTO) thin-film device with double layers of different oxygen density.

The device structure of the α-GTO thin-film device with double layers of different oxygen density is shown in Figure 1. First, a quartz glass substrate is prepared, and Al bottom electrodes are deposited using vacuum evaporation by electrical resistance heating through a metal mask to form horizontal bus lines, whose film thickness is 50 nm and line/space is 150/150 µm. Next, the double layers of the α-GTO thin films are successively deposited using radio-frequency (RF) magnetron sputtering with a ceramics target of Ga:Sn = 1:3, plasma power of 60 W, deposition pressure of 1 Pa, substrate temperature at room temperature, and RF frequency of 13.56 MHz, whose film thickness is 25 nm each. The lower layer is deposited using sputtering gas of Ar:O_2_ = 20:0 sccm, whereas the upper layer is done using that of Ar:O_2_ = 20:10 sccm. As a result, it is assumed that the lower layer is an oxygen-poor (O-poor) layer and contains more oxygen vacancies, whereas the upper layer is an oxygen-rich (O-rich) layer and contains fewer oxygen vacancies. Next, Al top electrodes are deposited to form vertical bus lines, whose film thickness is 50 nm and line/space is 150/150 µm. No additional annealing process is done. Finally, the α-GTO thin-film device has a device structure that the double sequentially-stacked layers of different oxygen density are sandwiched between the Al top and bottom electrodes, where the area of the α-GTO thin-film device is 150 × 150 µm equivalent to the cross point of the Al top and bottom electrodes.

## 3. Results

### 3.1. Electrical Characteristics

The electrical characteristics of the α-GTO thin films are shown in Table 1. Here, only the α-GTO thin films are deposited on the quartz glass substrates using the same abovementioned fabrication process, that is, an α-GTO thin film is deposited using sputtering gas of Ar:O_2_ = 20:0 sccm, whereas another α-GTO thin film is done using that of Ar:O_2_ = 20:10 sccm, and the Hall effect is measured. It was found that the electrical conductivity for Ar:O_2_ = 20:0 is quite higher than that for Ar:O_2_ = 20:10. The free carriers are electrons for both films, the carrier density is 7.74 × 10^17^ cm^−3^, and the Hall mobility is 10.9 cm^2^ V^−1^ s^−1^ for Ar:O_2_ = 20:0, and they cannot be determined for Ar:O_2_ = 20:10 because the electrical conductivity is too low.

### 3.2. X-Ray Photoelectron Spectroscopy Spectrums

**Figure 2:**
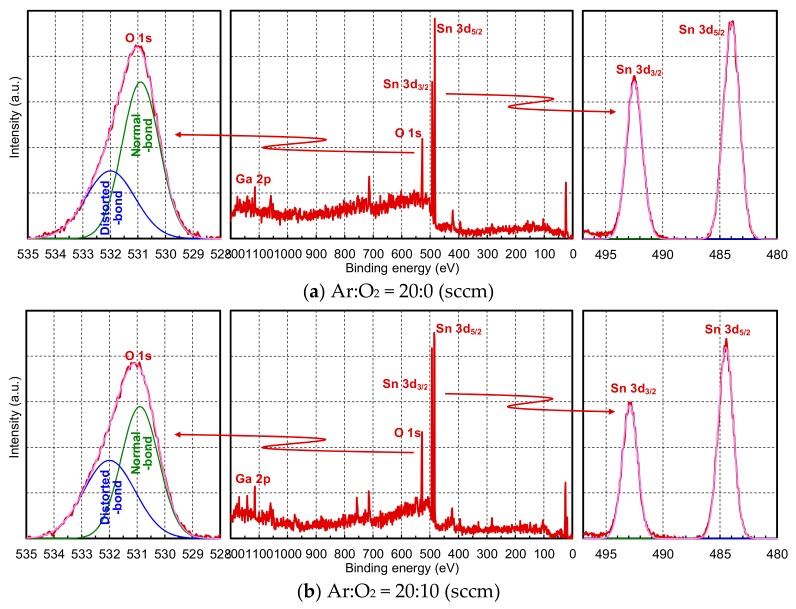
X-ray photoelectron spectroscopy (XPS) spectrums of the α-GTO thin films. (**a**) Ar:O_2_ = 20:0 (sccm), (**b**) Ar:O_2_ = 20:10 (sccm).

The X-ray photoelectron spectroscopy (XPS) spectrums of the α-GTO thin films are shown in Figure 2. Here, only the α-GTO thin films are deposited on the silicon wafer using the same abovementioned fabrication process. The Sn 3d_3/2_ and Sn 3d_5/2_ peaks are fitted with Gaussian distributions, and the O 1s peak is fitted with a sum of two Gaussian distributions, namely, that from oxygen normally bonded in the crystal lattice and that from oxygen distortedly bonded.

The relative elemental composition ratios of the α-GTO thin films are shown in Table 2. Here, O/Sn is defined as the ratio between the area under the O 1s peak and the sum of the areas under the Sn 3d_3/2_ and Sn 3d_5/2_ peaks, whereas distorted-bond/O is defined as the ratio between the area under the distorted-bond peak and that under the O 1s peak. It was found that the O/Sn for Ar:O_2_ = 20:0 is lower than that for Ar:O_2_ = 20:10. This means that the α-GTO thin film for Ar:O_2_ = 20:0 is an O-poor layer, whereas that for Ar:O_2_ = 20:10 is an O-rich layer. Moreover, it was also found that the distorted-bond/O for Ar:O_2_ = 20:0 is lower than that for Ar:O_2_ = 20:10. Although the distorted-bond peak is sometimes interpreted as an oxygen vacancy peak, it originally signals from oxygen distortedly bonded not only from oxygen related to oxygen vacancies but also oxygen between the crystal lattice; what can be said absolutely here is that the α-GTO thin film for Ar:O_2_ = 20:10 contains more oxygen. As a result, it is assumed that the α-GTO thin film for Ar:O_2_ = 20:0 contains more oxygen vacancies, whereas that for Ar:O_2_ = 20:10 contains fewer vacancies, which is consistent with the abovementioned electrical characteristics of the α-GTO thin films. In any case, as written above, it is assumed that the lower layer in the α-GTO thin-film device is an O-poor layer and contains more oxygen vacancies, whereas the upper layer is an O-rich layer and contains fewer oxygen vacancies.

### 3.3. Memristive Characteristic

**Figure 3:**
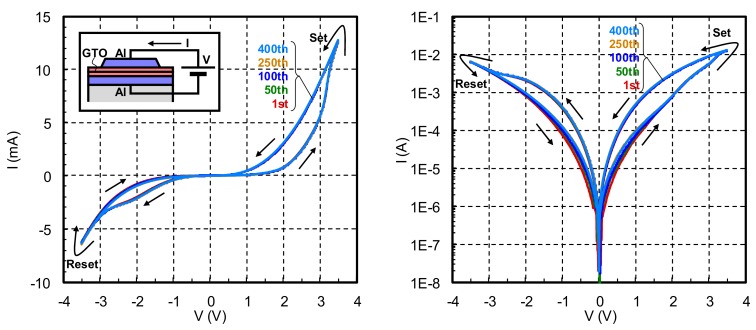
Memristive characteristic of the α-GTO thin film-device with double layers of different oxygen density.

The memristive characteristic of the α-GTO thin-film device with double layers of different oxygen density is shown in Figure 3. Here, voltage (V) is applied between the Al top and bottom electrodes and swept between −3.5 V and +3.5 V, and the electrical current (I) flows through the α-GTO thin-film device and is measured. It is found that as V rises from 0 V to +3.5 V, I also rises. When V falls from +3.5 V, |I| is larger than the previous |I|, which is called "set alternation". On the other hand, as |V| rises from 0 V to −3.5 V, |I| also rises. When |V| falls from −3.5 V, |I| is smaller than the previous |I|, which is called "reset alternation". It should be noted that in comparison with the previous results, the memristive characteristic is stable even from the initial hysteresis and completely overlapped at least to the 400th hysteresis.

### 3.4. Repetition Characteristic

**Figure 4:**
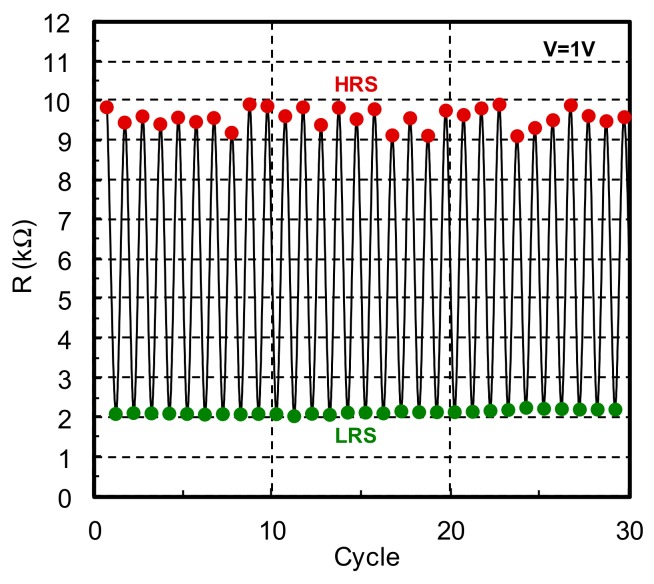
Repetition characteristic of the α-GTO thin-film device with double layers of different oxygen density.

The repetition characteristic of the α-GTO thin-film device with double layers of different oxygen density is shown in Figure 4. Here, the high-resistance state (HRS) is defined as a nonvolatile state after the reset alternation, whereas the low-resistance state (LRS) is defined as a nonvolatile state after the set alternation, and the electrical resistances are defined when |V| is +1 V for the HRS and LRS. It is found that the electrical resistances are clearly discretized for the HRS and LRS, and the switching ratio, namely, the resistance ratio between the HRS and LRS, is roughly 5.0. In any case, we have found a memristive characteristic of an α-GTO thin-film device with double layers of different oxygen density, which is surely stable from the initial hysteresis in comparison with the previous results.

## 4. Discussion

**Figure 5:**
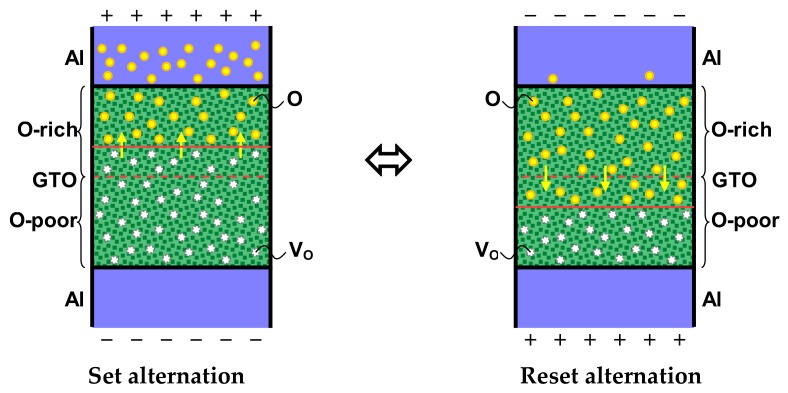
Operating mechanism of the memristive characteristic.

The operating mechanism of the memristive characteristic is shown in Figure 5. As aforementioned, it is assumed that the lower layer is an O-poor layer and contains more oxygen vacancies (V_O_), whereas the upper layer is an O-rich layer and contains fewer oxygen vacancies. The memristive characteristic can be explained by the drift of the oxygen in the α-GTO thin films through the interface of the double layers. When +3.5 V is applied to the Al top electrode for the set alternation, the oxygen is concentrated to the upper part of the α-GTO thin films, the region including the oxygen vacancies becomes thicker, and because the region has high electrical conductivity, |I| becomes larger. On the other hand, when −3.5 V is applied for the reset alternation, the oxygen is distributed to the lower part, the region including oxygen vacancies becomes thinner, and |I| becomes smaller. This operating mechanism is possible only when the double layers of different oxygen density are prepared.

The reason why the memristive characteristic is stable without the forming operation is because it is not necessary that some additional structure such as a filament is formed. Moreover, we believe that the behaviors of the oxygen between the O-poor and O-rich layers are fortunately similar from the initial hysteresis to the later equilibrium states. Incidentally, whereas AlO_x_ at the electrode interfaces plays a major role in the previous results [37], it is not important here because the memristive characteristic occurs at the interface of the double layers of different oxygen density. The operating mechanism is consistent with the fact that the memristive characteristic is the same when the lower and upper layers are upside down, that is, the lower layer is deposited using sputtering gas of Ar:O_2_ = 20:10 sccm, whereas the upper layer is done using that of Ar:O_2_ = 20:0 sccm, and the applied voltage is also upside down, which means that the memristive characteristic occurs at the interface of the double layers.

## 5. Conclusions

We have found a memristive characteristic of α-GTO thin-film device with double layers of different oxygen density. In comparison with the previous results, the memristive characteristic was stable even from the initial hysteresis. The double layers of the α-GTO thin films were successively deposited using RF magnetron sputtering. The sputtering gas for the deposition of the lower layer contained less oxygen, whereas that for the upper layer contained more oxygen. As a result, it was assumed that the lower layer contained more oxygen vacancies, whereas the upper layer contained fewer oxygen vacancies. The memristive characteristic was be able to be explained by the drift of the oxygen in the α-GTO thin films. Moreover, it should be noted that the memristive characteristic was stable without the forming operation because it was not necessary that some additional structure such as a filament was formed. It should be also noted that the fabrication process was not extended because the double layers were able to successively deposit simply by changing the oxygen ratio in the sputtering gas in the same vacuum chamber.

## Figures and Tables

**Table 1:** Electrical characteristics of the α-GTO thin films.

Ar:O_2_	(sccm)	20:0	20:10
Electrical conductivity	(S cm^−1^)	1.35	<10^−4^
Free carrier	(e^−^ or h^+^)	e^−^	e^−^
Carrier density	(cm^−3^)	7.74 × 10^17^	Determination impossible
Hall mobility	(cm^2^ V^−1^ s^−1^)	10.9	

**Table 2:** Relative elemental composition ratios of the α-GTO thin films.

Ar:O_2_	20:0	20:10
O/Sn (O 1s)/(Sn 3d_3/2_ + Sn 3d_5/2_)	0.220	0.259
Distorted-bond/O (Distorted-bond)/(O 1s)	0.575	0.814
